# The Potential of Trial-by-Trial Variabilities of Ongoing-EEG, Evoked Potentials, Event Related Potentials and fMRI as Diagnostic Markers for Neuropsychiatric Disorders

**DOI:** 10.3389/fnins.2018.00850

**Published:** 2019-07-17

**Authors:** Carlos Trenado, Anaí González-Ramírez, Victoria Lizárraga-Cortés, Nicole Pedroarena Leal, Elias Manjarrez, Diane Ruge

**Affiliations:** ^1^Translational Neuromodulation Unit, Department of Psychology and Neurosciences, Leibniz Research Centre for Working Environment and Human Factors, Technical University Dortmund, Dortmund, Germany; ^2^Integrative Neurophysiology and Neurophysics, Institute of Physiology, Benemérita Universidad Autónoma de Puebla, Puebla, Mexico

**Keywords:** trial-by-trial variability, trial-to-trial, single-trial, inter-trial, intertrial

## The “Trial-by-trial Variability” and Synonyms

The theme of “neuronal variability” is of great importance in the characterization of the “inter and intra-subject variability,” a concept that is becoming crucial to understand neuronal adaptation and plasticity in aging, and many pathological conditions of the brain. There are many comprehensive reviews about the “inter and intra-subject variability” of many behavioral and brain signals, in patients with neurological disorders and healthy controls (MacDonald et al., 2006, 2009; Faisal et al., 2008; Fontanini and Katz, 2008; Geurts et al., 2008; Vanrullen et al., 2011; Dykiert et al., 2012; Tamm et al., 2012; Garrett et al., 2013; Kofler et al., 2013; Dinstein et al., 2015; Dhawale et al., 2017; Ouyang et al., 2017; Tian et al., 2017; Haigh, 2018). However, there are no reviews focused on the “inter and intra-subject variability” for evoked potentials (EP) or event related potentials (ERP).

We found that many authors employed different terms to describe neuronal variability as: trial-by-trial, trial-to-trial, single-trial, inter-trial, intertrial. Also, many references are as old as the origin of the EEG technique, introduced by Berger (1929). For instance, Adrian and Yamagiwa (1935) noted that there are considerable variations in one subject and another regarding the fluctuation in size of the ongoing-EEG waves. In the same way, Barlow (1959), Davis and Zerlin (1964) studied the variability of EPs with the idea that such variability could serve to obtain normative data, which could explain behavioral and developmental observations in patients with neuropsychiatric disorders and healthy controls (see also Zerlin and Davis, 1967; Cigánek, 1969; Lifshitz, 1969; Callaway et al., 1970).

A constructive critic for the reviews mentioned in the first paragraph of this section is that the authors of such reviews did not cite relevant studies of trial-by-trial EP variability. For example, they did not cite the study by Callaway and Halliday (1973), who found that the variability of visual and auditory EPs decreased with increased age. They also ignored an essential study by Callaway and Halliday (1973), who were the first to perform a test and retest of the EPs separated by a year, as recently was done by Arazi et al. (2017). Another point no discussed in such reviews is that the results by Williams et al. (2005) are consistent with the pioneer findings of Callaway and Halliday (1973). In fact, Williams et al. (2005) found that the behavioral variability (choice reaction time) falls as age increases. In the same way, Callaway and Halliday (1973) found that the EP variability also falls as age increases. This means that both variabilities change as a function of age.

A possible explanation for these omissions of the old literature could be the indiscriminate use of different terms to refer to the “neuronal variability.” Another possible explanation for such omissions is the use of different synonyms for the terms “inter and intra-subject variability.” The intra-subject variability is the variability in behavior, or in any signal, of a single individual measured across multiple time points. In contrast, inter-individual variability is defined as the variability in behavior or in any signal exhibited by multiple individuals at a single time point.

As a conclusion of this section, we highlight that the terminology should be defined in consensus to quickly localize the literature in this field and guarantee a fluid advance of new questions and clinical applications of the trial-by-trial variability.

## The “Trial-by-trial Variabilities” of Ongoing-EEG, ERPs and fMRI Signals as Neuronal Markers of Neuropsychiatric Disorders. Origins and New Tendencies

Two pioneering studies motivated the analysis of the trial-by-trial variability of EPs. Barlow published the first in 1959, who invented an electronic device to detect the variance of EPs analogically. Zerlin and Davis published the second study in 1967, who presented compelling data from a subject with uniquely high amplitude auditory EPs (not averaged). Such reports, supported the fact that it is possible to obtain normative data for the variability of EPs, even that they only could be detected by averaging techniques (Cigánek, 1968), or by multiple linear regression approaches in the trial-by-trial analysis (Iannetti et al., 2005, 2006). In fact, Iannetti et al. (2005, 2006) also recorded EPs (not averaged) as Zerlin and Davis (1967). They were able to elicit trial-by-trial EPs related to a brief painful stimulus with a laser. The laser pulses excited Aδ and C nociceptors in the superficial skin layers and they produced a high amplitude response in the brain, which was processed with multiple linear regression approaches in the trial-by-trial analysis.

Jones et al. (1965), Marjerrison et al. (1968), Lifshitz (1969), and Callaway et al. (1970) published other pioneering studies, reporting that the EP variability correlates with neuropsychiatric disorders, behavior, and aging. Jones et al. (1965), Marjerrison et al. (1968), Lifshitz (1969), and Callaway et al. (1970) found that the variability of EPs is high both in schizophrenia and in young children. These seminal reports motivated the use of the trial-by-trial analysis of the EPs as a potential neuronal marker of disease, and they were a preamble for the analysis of behavioral variability across the lifespan (see review in MacDonald et al., 2006). In the case of schizophrenia, many studies reported that these patients tend to have smaller and more variable EPs than healthy subjects (Jones and Callaway, 1970; Saletu et al., 1971; Borge, 1973; Cohen, 1973; Rappaport et al., 1975; Buchsbaum and Coppola, 1979; Winterer and Weinberger, 2004; Jansen et al., 2010; Shin et al., 2015). Consistently, there is also a greater trial-by-trial variability in autism than in healthy controls (Milne, 2011; Dinstein et al., 2012; Haigh et al., 2014).

Furthermore, the concurrent or separated trial-by-trial analysis of the ongoing-EEG, EPs, ERPs and fMRI could be the future in the field of the inter- and intra-subject variability, in patients with schizophrenia, autism, and other neuropsychiatric disorders. For instance, Haigh et al. (2017) examined differential sensory fMRI signatures in autism and schizophrenia via an analysis of amplitude and trial-by-trial variability. These authors found that autism was associated with more considerable trial-by-trial variability, whereas schizophrenia was associated with smaller response amplitudes. Future studies will be necessary to explain whether the trial-by-trial variation in fMRI signatures could be used as a reliable biomarker for schizophrenia as the trial-by-trial analysis of EPs.

In this context, there are many reports obtained from healthy subjects that could be replicated in patients with neuropsychiatric disorders. In this paragraph, we describe some of them in chronological order. For example, Debener et al. (2005) demonstrated that the single trial-by-trial error-related negativity of the EEG reflects quick behavioral adjustments that predict the fMRI activity of brain regions engaged in the processing of response errors. Mantini et al. (2009), employed the trial-by-trial EEG/fMRI correlation approach and ERPs during target detection, to show that the ventral attention network was transiently activated by the occurrence of targets, while the dorsal attention network reflects sustained activity. In a similar way, Goldman et al. (2009), employed the trial-by-trial analysis of the combined use of ongoing-EEG, ERPs and fMRI to identify cortical areas associated with EEG components related to an oddball auditory paradigm. Also, Hesselmann et al. (2008), used the trial-by-trial analysis of the ongoing-EEG, ERPs, and fMRI to show that the fMRI signals in the inferior parietal lobe and precentral gyrus, exhibit a significant covariation with the P3 related activity. In another study, Mayhew et al. (2013), performed the simultaneous ongoing-EEG/fMRI recording during rest and during the noxious thermal stimulation, discovering an interesting interaction between the alpha-power, the fMRI activity, and the trial-by-trial variability of the behavioral report of pain. Other studies also combined the analysis of EEG/fMRI signals with the trial-by-trial variability in the context of neuronal connectivity (Goltz et al., 2015) and working memory maintenance (Scheeringa et al., 2016). According to Gluth and Rieskamp (2017), the trial-by-trial variability is a useful way to link variance in behavior that cannot be exclusively explained by cognitive modeling with ongoing-EEG, ERPs, and fMRI.

Therefore, based on the abovementioned references, we could mention three essential aspects that highlight the importance of the trial-by-trial variability analysis of ongoing-EEG, ERPs, and fMRI responses of the impaired brain. First, the increased variability in the behavioral and trial-by-trial ERPs is a universal feature in patients with schizophrenia and autism. Second, it makes possible the analysis of the relationships in the variation among the ongoing-EEG-prestimulus brain-states, the trial-by-trial ERPs, and the associated behavioral outcomes (De Blasio and Barry, 2018). This second aspect is particularly important because the study of such variation across the life-span may allow exploring mechanisms of healthy and pathological aging (De Blasio and Barry, 2018). Third, the combined use of ongoing-EEG, EPs, ERPs and fMRI signals with their trial-by-trial variability analysis could be of great importance in the mechanistic exploration of neuropsychiatric disorders.

As a conclusion of this section, we highlight that the trial-by-trial variability analysis of ongoing-EEG, EPs, ERPs, and fMRI signals could be considered as a potential neuronal marker for neuropsychiatric disorders, as schizophrenia, Alzheimer disease, depression, borderline personality disorder, autism, and attention deficit hyperactivity disorder (Schwartz et al., 1989; Hogan et al., 2006; Geurts et al., 2008; Kaiser et al., 2008; Dinstein et al., 2015; Saville et al., 2015).

## Limitations and Recommendations for the Analysis of Trial-by-trial Variability of EEG/fMRI Signals

A limitation of the use of the trial-by-trial variability as a diagnostic marker includes the requirement of a high number of single trials to extract reliable ERPs and behavioral patterns (Boudewyn et al., 2018). On the other hand, most of the patients are medicated and this could add a possible bias in the data interpretation. Castellanos et al. (2005) and Kaiser et al. (2008), mention that a serious confound may arise from the dopamine receptor blocking medication in patients with schizophrenia, because an impairment of catecholaminergic neurotransmission has been postulated as a cause of increased intra-subject variability. Another limitation is that we know little about the physiological mechanisms for the relationships between the trial-by-trial variability of the ongoing-EEG, EPs, ERPs, fMRI, and behavior. Future work will be necessary to disentangle such relationships in patients with diverse neuropsychiatric disorders and healthy controls (MacDonald et al., 2006; Kaiser et al., 2008). Another limitation is the cooperation of the same subjects in the EEG aging paradigms across the lifespan. For instance, it would be complicated to obtain a similar U-like curve for the trial-by-trial EPs, as the curve obtained by Williams et al. (2005) for the reaction time across the lifespan (see also MacDonald et al., 2006). Such type of studies would require the development of new wearable EEG systems that could be used across the lifespan (Tian et al., 2019). Such systems would require new technologies for the development of low power, small size, and imperceptible weight, and they are in state of the art for wearable EEG solutions.

Because in the literature there are many terms for the “trial-by-trial variability” and the “inter and intra-subject” variability, we recommend the standardized use of these terms in future publications and a consensus statement. In the same context, we recommend the writing of a systematic review and meta-analysis about this issue, following similar methods as Dykiert et al. (2012).

## Author Contributions

All authors listed have made a substantial, direct and intellectual contribution to the work, and approved it for publication.

### Conflict of Interest Statement

The authors declare that the research was conducted in the absence of any commercial or financial relationships that could be construed as a potential conflict of interest.
